# Utilization of Transcriptome, Small RNA, and Degradome Sequencing to Provide Insights Into Drought Stress and Rewatering Treatment in *Medicago ruthenica*

**DOI:** 10.3389/fpls.2021.675903

**Published:** 2021-08-03

**Authors:** Rui Shi, Wei Jiao, Lan Yun, Zhiqiang Zhang, Xiujuan Zhang, Quanzhen Wang, Ying Li, Fugui Mi

**Affiliations:** ^1^Key Laboratory of Grassland Resources, Ministry of Education P.R of China, College of Grassland, Resources and Environment, Inner Mongolia Agricultural University, Hohhot, China; ^2^Baotou Medical College, Baotou, China; ^3^College of Mechanical and Electrical Engineering, Inner Mongolia Agricultural University, Hohhot, China; ^4^Institute of Grassland Research, Chinese Academy of Agricultural Sciences, Hohhot, China; ^5^Inner Mongolia Key Laboratory of Molecular Biology on Featured Plants, Hohhot, China; ^6^College of Grassland Agriculture, Northwest A&F University, Yangling, China

**Keywords:** *Medicago ruthenica*, drought, differential expression, miRNA, degradome

## Abstract

Drought is a major limiting factor in foraging grass yield and quality. *Medicago ruthenica* (*M. ruthenica*) is a high-quality forage legume with drought resistance, cold tolerance, and strong adaptability. In this study, we integrated transcriptome, small RNA, and degradome sequencing in identifying drought response genes, microRNAs (miRNAs), and key miRNA-target pairs in *M. ruthenica* under drought and rewatering treatment conditions. A total of 3,905 genes and 50 miRNAs (45 conserved and 5 novel miRNAs) were significantly differentially expressed in three test conditions (CK: control, DS: plants under drought stress, and RW: plants rewatering after drought stress). The degradome sequencing (AllenScore < 4) analysis revealed that 104 miRNAs (11 novel and 93 conserved miRNAs) were identified with 263 target transcripts, forming 296 miRNA-target pairs in three libraries. There were 38 differentially expressed targets from 16 miRNAs in DS vs. CK, 31 from 11 miRNAs in DS vs. RW, and 6 from 3 miRNAs in RW vs. CK; 21, 18, and 3 miRNA-target gene pairs showed reverse expression patterns in DS vs. CK, DS vs. RW, and RW vs. CK comparison groups, respectively. These findings provide valuable information for further functional characterization of genes and miRNAs in response to abiotic stress, in general, and drought stress in *M. ruthenica*, and potentially contribute to drought resistance breeding of forage in the future.

## Introduction

Drought is the most widespread climatic extreme that has a negative impact on human and natural environments (Touma et al., 2015; Schwalm et al., 2017). It has received more attention with the increase of severe drought occurrences. The physiological acclimatization of individuals may buffer the effect of drought (Schwalm et al., 2017); thus, it is meaningful to improve adaptability in plants by increasing their drought stress tolerance.

Plants have specific adaptive responses that protect them from environmental stresses (Chinnusamy et al., 2004), which are generally controlled by many complex regulatory networks involving numerous genes. The regulation of gene expression can be carried out at the transcriptional, post-transcriptional, and epigenetic modification levels. In plants, several biological processes are regulated by small RNAs at the transcriptional and post-transcriptional levels, including plant growth and development processes, as well as biotic and abiotic stress responses (Trindade et al., 2010; Capitão et al., 2011; Liu et al., 2014; Gao et al., 2016; Shu et al., 2016; Garg et al., 2019). Small RNAs (21–26 nt) are ubiquitous, versatile repressors of gene expression in plants, animals, and many fungi, which include short interfering RNAs (siRNAs), small temporal RNAs (stRNAs), heterochromatic siRNAs, tiny non-coding RNAs, and microRNAs (miRNAs) (Finnegan and Matzke, 2003). In plants, miRNAs are endogenous non-coding small RNAs measuring 20–24 nt long (Jones-Rhoades et al., 2006). They negatively regulate gene expression at the post-transcriptional level *via* direct cleavage of the target mRNA or inhibition of target gene translation by recognizing and combining to their target mRNAs (Bartel, 2004; Voinnet, 2009). A single miRNA can have several target genes, and several miRNAs can regulate one gene. Therefore, the gene pool regulated by miRNAs can be very extensive.

Plant miRNAs are frequently complementary to their target mRNAs; this complementarity effectively triggers target mRNA degradation (Llave et al., 2002; Tang et al., 2003) and loss of protein-coding function (German et al., 2008; Iwakawa and Tomari, 2015). Most miRNAs regulate the expression of target genes in plants *via* splicing, and slicing often occurs at the tenth or eleventh nucleotide of the complementary region of the miRNA and mRNA. Thus, the identification of target genes is crucial for miRNA functional analysis. High-throughput sequencing is the most popular technique for identifying miRNAs in plants, because it allows for an easier and faster access to large numbers of miRNAs, especially those of low abundance (Allen et al., 2004). Afterwards, the functions of miRNAs may be identified through bioinformatic prediction or degradome sequencing. Degradome sequencing screens out the target genes for miRNAs by combining the advantages of high-throughput sequencing technology and bioinformatics analysis. The key point of the correlation analysis of the transcriptome and small RNA is the result of the degradome sequencing.

*Medicago ruthenica* L. is a cross-pollinated, diploid (2*n* = 16) perennial legume forage, with a remarkable ability to adapt to extreme environments. Thus, *M. ruthenica* is considered a valuable forage crop, which may be used as a potential source of genes to improve abiotic stress resistance in cultivated alfalfa (Campbell et al., 1997). There have been many reports on the molecular mechanisms induced by drought stress in leguminous forage (Li et al., 2017; Zhang et al., 2018, 2019), but few on *M. ruthenica*. Moreover, a number of miRNAs have been identified to be associated with responses to drought stress in several species, such as *Arabidopsis thaliana* (Liu et al., 2008), tomato (Liu et al., 2018), *Oryza sativa* (Chen and Li, 2018; Nadarajah and Kumar, 2019), and *Gossypium hirsutum* (Lu et al., 2019). Here, we integrated transcriptome, miRNAome, and degradome results to identify drought-response genes and miRNAs in *M. ruthenica*, and find potential regulatory patterns of miRNA-target pairs. These results may provide novel insights into the response to abiotic stresses of *M. ruthenica*, and contribute to drought resistance breeding of forage in the future.

## Results

### Transcriptome Sequencing in *M. ruthenica* Under Drought Stress

To profile the expression of genes in *M. ruthenica* in response to drought stress, nine libraries were constructed from three leaf samples (CK: control, DS: plants under drought stress, RW: plants rewatering after drought stress), each with three biological replicates. Then, 36,207,356 to 55,818,774 raw data were generated, accounting for 5.43–8.37 GB of sequencing data (Supplementary Table 1). A total of 147,957 transcripts were obtained from all cDNA libraries. After conducting quality control, the transcripts were assembled into 52,457 unigene clusters, with an N50 value of 1,451 bp. A summary of the transcriptome sequencing of *M. ruthenica* is shown in Table 1.

**TABLE 1:** Summary of *M. ruthenica* transcriptome sequencing.

Index	Transcript	Gene
All	147,957	52,457
GC%	39.48	39.19
Min length (bp)	201	201
Median length (bp)	582	497
Max length (bp)	15,683	15,683
Total assembled bases	128,392,014	44,936,112
N50 (bp)	1,323	1,451

All assembled unigene clusters were aligned against Gene Ontology (GO),^1^ Kyoto Encyclopedia of Genes and Genomes (KEGG),^2^ Pfam database,^3^ SwissProt database,^4^ evolutionary genealogy of genes: Non-supervised Orthologous Groups (eggNOG)^5^ databases, and NCBI non-redundant protein database (NCBI_NR)^6^ using DIAMOND 23 with a threshold *E*-value < 0.00001 (Buchfink et al., 2015). The statistical results from six authoritative databases are listed in Table 2.

**TABLE 2:** Statistical results from the DIAMOND 23 annotation.

DB	Number	Ratio (%)
All	52,457	100.00
GO	25,074	47.80
KEGG	20,934	39.91
Pfam	23,700	45.18
SwissProt	20,598	39.27
eggNOG	28,833	54.97
NR	33,136	63.17

### Differentially Expressed Genes in *M. ruthenica* Under Drought Stress

**FIGURE 1:**
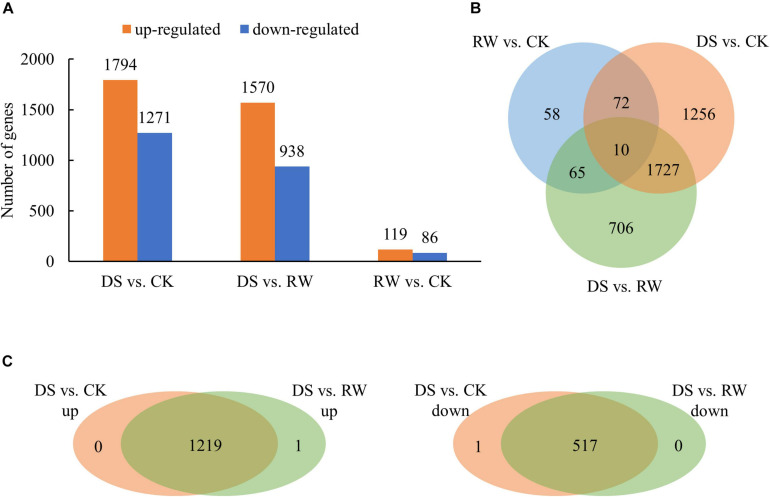
Differentially expressed gene analysis for control (CK), drought stress (DS), and rewatering (RW) treatment in *M. ruthenica*. **(A)** The number of DEGs for the DS vs. CK, DS vs. RW, and RW vs. CK comparisons (*p* < 0.05); orange, upregulated DEGs; blue, downregulated DEGs. **(B)** Venn diagram of overlapping DEGs among the three comparisons. **(C)** Venn diagrams to illustrate the overlapping DEGs with upregulated expression between the DS vs. CK comparison and the DS vs. RW comparison, and the overlapping DEGs with downregulated expression.

To identify the differentially regulated genes under drought stress in *M. ruthenica*, three comparisons of the three test conditions (CK, DS, and RW) were performed. In total, 3,905 genes were significantly differentially expressed among the three test groups. There were 3,065, 2,508, and 205 differentially expressed genes (DEGs) in the DS vs. CK, DS vs. RW, and RW vs. CK comparisons, respectively (Figure 1A). The overlapping DEGs among the three comparisons were shown in a Venn diagram in Figure 1B. The DEG overlap (1,737 overlapping DEGs) between DS vs. CK and DS vs. RW was much greater than the other two comparison groups (82 overlapping DEGs between DS vs. CK and RW vs. CK, 75 DEGs between DS vs. RW and RW vs. CK). Further analysis of the 1,737 overlapping DEGs showed that 1,219 upregulated genes and 517 downregulated genes were exactly the same in the DS vs. CK and DS vs. RW comparisons, except for one DEG (TRINITY_DN21640_c0_g2), which exhibited opposing trends (Figure 1C); this indicates that the 1,736 overlapping DEGs were involved in drought stress responses in *M. ruthenica*. Among all DEGs, 10 showed differential expression across all the treatments (Figure 1B). The information of all the DEGs in three comparisons is shown in Supplementary Table 2.

### Functional Annotation and Enrichment Analysis of the DEGs

In total, 2,866 DEGs were annotated to 2,186 GO terms *via* GO analysis. We detected significant enrichments (*p* < 0.05) of 331 GO terms in *M. ruthenica* under drought stress (Supplementary Table 3). In the “biological processes” category, the main terms, including “regulation of transcription, DNA-templated” (GO:0006355), “transcription, DNA-templated” (GO:0006351), “protein phosphorylation” (GO:0006468), and “oxidation-reduction process” (GO:0055114), were highly enriched in the DEGs; In the “molecular function” category, DEGs were significantly enriched in the terms “molecular function” (GO:0003674), “DNA binding transcription factor activity” (GO:0003700), “DNA binding” (GO:0003677), “protein serine/threonine kinase activity” (GO:0004674), and “sequence-specific DNA binding” (GO:0043565); In the “cellular component” category, DEGs were significantly enriched in the terms “plasma membrane” (GO:0005886), “integral component of membrane” (GO:0016021), “chloroplast” (GO:0009507), “membrane” (GO:0016020), and “extracellular region” (GO:0005576).

Then, with KEGG analysis, 2,201 DEGs were annotated to 122 different pathways, of which, 29 were significantly enriched (*p* < 0.05) (Supplementary Table 3). The DEGs were mostly included in the KEGG pathways of “Plant hormone signal transduction,” “Plant–pathogen interaction,” and “MAPK signaling pathway – plant,” indicating their significant roles during drought stress.

Table 3 showed the DEGs that are involved in two key KEGG pathways. The DEGs that enriched both in ko04075 (plant hormone signal transduction) and ko04016 (MAPK signaling pathway – plant) pathways included genes like transcription factor bHLH, transcription factor MYC, serine/threonine-protein kinase, abscisic acid receptor PYL, and probable protein phosphatase 2C. The DEGs that enriched both in ko04626 (plant–pathogen interaction) and ko04016 (MAPK signaling pathway – plant) pathways included genes like WRKY transcription factor, hypothetical protein TSUD, LRR receptor-like kinase family protein, LRR receptor-like kinase resistance protein, and calmodulin.

**TABLE 3:** The DEGs associated with ko04075 (plant hormone signal transduction), ko04626 (plant–pathogen interaction), and ko04016 (MAPK signaling pathway – plant) pathways.

Gene_ID	Annotation	Name
**The DEGs associated with ko04075 and ko04016 pathways**		
TRINITY_DN22772_c2_g1	Abscisic acid receptor PYL4 (*Medicago truncatula*)	PYL4
TRINITY_DN14995_c0_g6	Abscisic acid receptor PYL4 (*Medicago truncatula*)	PYL4
TRINITY_DN24297_c0_g1	Basic helix loop helix (BHLH) family transcription factor (*Medicago truncatula*)	MYC2
TRINITY_DN23594_c1_g1	EIN3-binding F-box protein 1 (*Medicago truncatula*)	EBF2
TRINITY_DN23245_c0_g1	EIN3-binding F-box protein 1 (*Medicago truncatula*)	EBF2
TRINITY_DN12343_c0_g1	Ethylene response factor 5 (*Medicago sativa*)	ERF1B
TRINITY_DN12343_c0_g2	Ethylene-responsive transcription factor 1B (*Medicago truncatula*)	ERF1B
TRINITY_DN20251_c0_g2	Hypothetical protein TSUD_14780 (*Trifolium subterraneum*)	PYL8
TRINITY_DN16118_c1_g2	PREDICTED: serine/threonine-protein kinase SAPK1-like isoform X2 (*Lupinus angustifolius*)	SAPK2
TRINITY_DN22472_c0_g2	Probable protein phosphatase 2C 51 (*Medicago truncatula*)	Os05g0572700
TRINITY_DN22472_c0_g4	Probable protein phosphatase 2C 51 (*Medicago truncatula*)	Os05g0572700
TRINITY_DN19234_c0_g2	Putative protein-serine/threonine phosphatase (*Medicago truncatula*)	PP2CA
TRINITY_DN25407_c0_g1	Putative protein-serine/threonine phosphatase (*Medicago truncatula*)	SAG113
TRINITY_DN25293_c1_g2	Putative reverse transcriptase, RNA-dependent DNA polymerase (*Medicago truncatula*)	PRB1
TRINITY_DN14527_c0_g1	Putative reverse transcriptase, RNA-dependent DNA polymerase (*Medicago truncatula*)	–
TRINITY_DN20934_c0_g2	Putative transcription factor bHLH family (*Medicago truncatula*)	AIB
TRINITY_DN15293_c0_g1	Receptor like protein kinase S.2 (*Medicago truncatula*)	LECRKS2
TRINITY_DN16118_c0_g1	Serine/threonine-protein kinase SAPK2 (*Medicago truncatula*)	SAPK2
TRINITY_DN18125_c0_g2	Serine/threonine-protein kinase SRK2A-like protein (*Trifolium pretense*)	SRK2H
TRINITY_DN19936_c0_g1	Serine/threonine-protein kinase SRK2E (*Medicago truncatula*)	SRK2E
TRINITY_DN17078_c0_g4	Transcription factor bHLH13 (*Medicago truncatula*)	BHLH13
TRINITY_DN20934_c0_g3	Transcription factor bHLH13 (*Medicago truncatula*)	BHLH13
TRINITY_DN22485_c0_g3	Transcription factor bHLH18 (*Medicago truncatula*)	BHLH25
TRINITY_DN25067_c1_g3	Transcription factor bHLH18 (*Medicago truncatula*)	BHLH25
TRINITY_DN23532_c1_g1	Transcription factor bHLH18 (*Medicago truncatula*)	BHLH25
TRINITY_DN25067_c1_g1	Transcription factor bHLH18 (*Medicago truncatula*)	BHLH25
TRINITY_DN19283_c0_g1	Transcription factor bHLH19 (*Medicago truncatula*)	BHLH25
TRINITY_DN25988_c1_g1	Transcription factor MYC2 (*Medicago truncatula*)	MYC2
TRINITY_DN17929_c0_g2	Transcription factor MYC2 (*Medicago truncatula*)	BHLH14
**The DEGs associated with ko04626 and ko04016 pathways**		
TRINITY_DN17467_c0_g4	Calmodulin (*Medicago truncatula*)	–
TRINITY_DN12142_c0_g1	EF-hand pair protein (*Medicago truncatula*)	–
TRINITY_DN19984_c0_g1	Esterase AGAP003155 isoform X1 (*Medicago truncatula*)	SPAC22A12.06c
TRINITY_DN19796_c0_g1	Hypothetical protein TSUD_205570 (*Trifolium subterraneum*)	WRKY11
TRINITY_DN22374_c0_g2	Hypothetical protein TSUD_302020 (*Trifolium subterraneum*)	AtMg01250
TRINITY_DN22688_c0_g2	Hypothetical protein TSUD_385260 (*Trifolium subterraneum*)	XA21
TRINITY_DN15050_c0_g1	LRR receptor-like kinase family protein (*Medicago truncatula*)	XA21
TRINITY_DN20801_c0_g3	LRR receptor-like kinase resistance protein, partial (*Trifolium pratense*)	–
TRINITY_DN13721_c0_g1	LRR receptor-like kinase resistance protein, partial (*Trifolium pratense*)	At3g47570
TRINITY_DN16445_c0_g3	LRR receptor-like serine/threonine-protein kinase FLS2 (*Medicago truncatula*)	FLS2
TRINITY_DN20143_c0_g1	MDIS1-interacting receptor like kinase 2 (*Medicago truncatula*)	PGIP2
TRINITY_DN15715_c0_g3	Probable calcium-binding protein CML45 (*Medicago truncatula*)	CML45
TRINITY_DN16518_c0_g2	Probable WRKY transcription factor 15 isoform X1 (*Medicago truncatula*)	WRKY15
TRINITY_DN25387_c0_g7	Probable WRKY transcription factor 28 (*Medicago truncatula*)	WRKY28
TRINITY_DN23864_c3_g1	Probable WRKY transcription factor 29 (*Medicago truncatula*)	WRKY29
TRINITY_DN18166_c0_g2	Probable WRKY transcription factor 33 (*Medicago truncatula*)	WRKY33
TRINITY_DN15895_c0_g2	Probable WRKY transcription factor 35 (*Medicago truncatula*)	WRKY35
TRINITY_DN24400_c0_g11	Probable WRKY transcription factor 47 (*Medicago truncatula*)	WRKY42
TRINITY_DN15622_c0_g12	Probable WRKY transcription factor 50 (*Medicago truncatula*)	WRKY50
TRINITY_DN20789_c0_g3	Probable WRKY transcription factor 65 (*Medicago truncatula*)	WRKY69
TRINITY_DN15214_c0_g1	Probable WRKY transcription factor 69 (*Medicago truncatula*)	WRKY69
TRINITY_DN14689_c0_g2	Putative protein kinase RLK-Pelle-LRR-XI-1 family (*Medicago truncatula*)	–
TRINITY_DN25293_c1_g2	Putative reverse transcriptase, RNA-dependent DNA polymerase (*Medicago truncatula*)	PRB1
TRINITY_DN14527_c0_g1	Putative reverse transcriptase, RNA-dependent DNA polymerase (*Medicago truncatula*)	–
TRINITY_DN14614_c0_g1	Putative RNA-directed DNA polymerase (*Medicago truncatula*)	AtMg00810
TRINITY_DN15293_c0_g1	Receptor like protein kinase S.2 (*Medicago truncatula*)	LECRKS2
TRINITY_DN17908_c0_g4	Respiratory burst oxidase homolog protein E (*Medicago truncatula*)	RBOHE
TRINITY_DN15806_c0_g5	Toll/interleukin-1 receptor-like protein (*Medicago truncatula*)	–
TRINITY_DN20306_c1_g5	Unnamed protein product, partial (*Brassica oleracea*)	CAM-1
TRINITY_DN20427_c1_g4	WRKY transcription factor WRKY108715 (*Medicago truncatula*)	WRKY24
TRINITY_DN15802_c1_g3	WRKY transcription factor WRKY24 isoform X1 (*Medicago truncatula*)	WRKY24
TRINITY_DN15802_c1_g2	WRKY transcription factor WRKY24 isoform X1 (*Medicago truncatula*)	WRKY24

### qRT-PCR Verification of mRNA-Seq Analysis of Gene Expression

**FIGURE 2:**
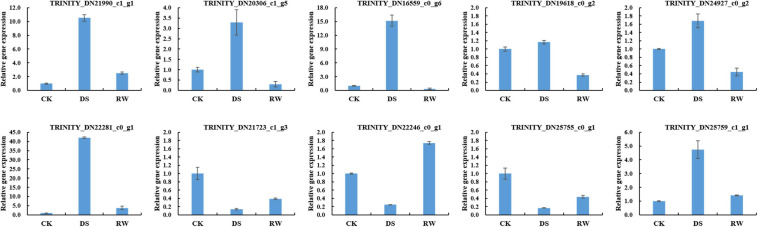
Relative gene expression for qRT-PCR verification of mRNA-Seq results. The data were the average of three qRT-PCR replicates for each sample from three biological replicates. *GAPDH* of alfalfa was used as an internal reference. Error bars indicate the standard deviation of three biological replicates.

To validate the mRNA-Seq results, 10 genes were selected for quantitative real-time polymerase chain reaction (qRT-PCR) analysis. The information of these genes is shown in Supplementary Table 4. The qRT-PCR results (Figure 2) showed similar expression trends to their high-throughput sequencing analysis, which suggested that our RNA-seq data are credible.

### Deep Sequencing of *M. ruthenica* Small RNAs

The small RNA deep sequencing of *M. ruthenica* was performed from three samples (CK, DS, and RW), each with three biological replicates. We counted the raw data sequencing outputs, and generated unique sequences and their corresponding copies. A total of 16,112,539, 13,520,602, and 17,459,447 reads, and 4,029,151, 2,602,204, and 3,314,079 unique sequences were generated from CK, DS, and RW, respectively (Supplementary Table 5). Meanwhile, by comparing the acquired sgRNA sequence with the mRNA, RFam (including rRNA, tRNA, snRNA, and snoRNA), and Repbase databases, we established 12,257,093, 6,163,546, and 10,663,527 valid reads, and 3,422,624, 1,675,939, and 2,468,514 valid unique sequences in the CK, DS, and RW groups. These valid data were subjected to further miRNA comparison identification and prediction analysis.

After removing the low-quality sequences, 18–25 nt long sequences were obtained. As shown in Table 4, 77.31% were 21- or 24-nt long unique miRNA sequences, which accounted for most classes of *M. ruthenica* sRNA. These results are consistent with those of other plant species, such as *A. thaliana* (Rajagopalan et al., 2006; Fahlgren et al., 2007), *Medicago truncatula* (Szittya et al., 2008; Eyles et al., 2013), and *Arachis hypogaea* (Chi et al., 2011).

**TABLE 4:** Length distribution of unique miRNAs.

Length (bp)	Unique miRNA	% Unique miRNA
18	21	3.53
19	15	2.52
20	11	1.85
21	184	30.92
22	61	10.25
23	21	3.53
24	276	46.39
25	6	1.01
All	595	100.00

### Identification of Conserved and Novel miRNAs in *M. ruthenica*

To identify conserved miRNAs in *M. ruthenica*, we compared the small RNA sequences with known plant miRNAs in the miRBase. Based on sequence homology, 532 conserved miRNAs belonging to 65 miRNA families, and 63 novel miRNAs were identified from the nine libraries (Table 5).

**TABLE 5:** Summary of identified conserved and novel miRNAs.

Groups	Pre-miRNA	Unique miRNA
gp1	37	56
gp2a	34	42
gp2b	365	359
gp3	67	75
gp4	57	63
Total	560	595

*gp1: Reads were mapped against the miRNAs/pre-miRNAs of specific species in miRBase, and the pre-miRNAs were further mapped to the genome and EST; gp2a: Reads were mapped against the miRNAs/pre-miRNAs of selected species in miRBase. The reads (as well as the miRNAs of the pre-miRNAs) were mapped against the genome, while the mapped pre-miRNAs were not. The extended genome sequences from the genome loci may form hairpins; gp2b: Reads were mapped against the miRNAs/pre-miRNAs of selected species in miRBase. The reads (as well as the miRNAs of the pre-miRNAs) were mapped to the genome, while the mapped pre-miRNAs were not. The extended genome sequences from the genome loci may not form hairpins; gp3: Reads were mapped against the miRNAs/pre-miRNAs of selected species in miRBase. The mapped pre-miRNAs and the reads were not mapped against the genome. However, the reads were mapped against the miRNAs (Matures); gp4: Reads were not mapped to pre-miRNAs of selected species in miRBase. However, the reads were mapped against the genome. The extended genome sequences may form hairpins.*

### Drought-Responsive miRNAs in *M. ruthenica*

**FIGURE 3:**
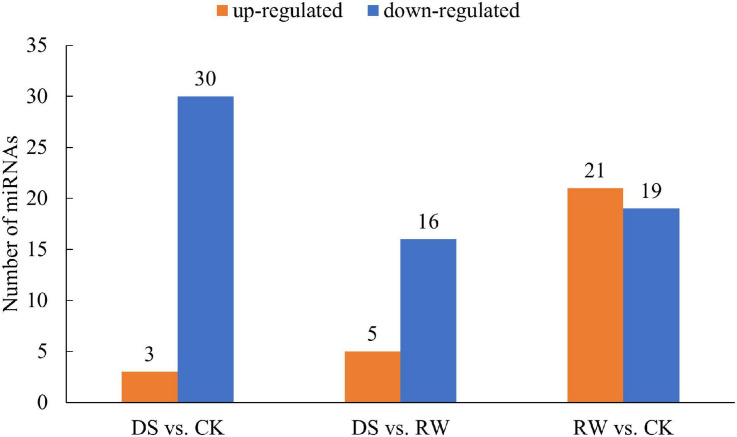
The number of differentially expressed miRNAs for the three comparisons (*p* < 0.05); orange, upregulated miRNA expression; blue, downregulated miRNA expression.

To identify miRNAs in response to drought in *M. ruthenica*, we analyzed and compared the read counts in nine libraries. miRNAs with a *p*-value < 0.05 were considered DEMs. In total, 33, 21, and 40 miRNAs were differentially expressed in the DS vs. CK, DS vs. RW, and RW vs. CK comparisons, respectively. Meanwhile, respective up- and downregulation profiles were found in each comparison: 3 and 30 miRNAs in DS vs. CK, 5 and 16 in DS vs. RW, and 21 and 19 in RW vs. CK (Figure 3). The details of these DEMs found in the three comparisons are shown in Supplementary Table 6.

Our results also revealed that 50 miRNAs showed significant differentially expressed patterns among the RW vs. DS vs. CK comparison, including 45 conserved miRNAs and 5 novel miRNAs (Table 6). Among the 50 DEMs, the expression of 23 miRNAs, such as gma-miR171j-5p, lja-miR390a-3p, mtr-miR398a-5p, and bra-MIR408-p5, was downregulated, and then upregulated during drought stress and rehydration, respectively. On the other hand, the expression of 12 miRNAs, such as mtr-miR156b-5p, mtr-MIR156e-p3, gma-miR159a-3p, and lus-miR396b, exhibited the opposite expression profiles during the same respective conditions. Moreover, 12 miRNAs, such as mtr-miR396a-5p, mtr-miR398a-3p, mtr-miR398b, mtr-miR408-3p, were always downregulated, and three miRNAs (gma-miR6300, gma-MIR5368-p5, and peu-MIR2916-p3) were upregulated during the entire experiment.

**TABLE 6:** The small RNA sequencing results of 50 DEMs in the CK, DS, and RW groups.

Index	miRNA name	CK	DS	RW	*p*-Value
1	gma-MIR10405a-p5_2ss8AT21TG	8	0	17	9.55E−05
2	mtr-miR398a-3p_L + 1R-1	869	68	35	1.40E−04
3	mtr-MIR5230-p3_2ss7TG19AG	9	0	11	7.63E−04
4	gma-miR6300_R + 5	78	218	433	8.14E−04
5	mtr-miR5299_L + 1R-1	2	0	5	2.17E−03
6	mtr-MIR2610a-p3_1ss9AC	7	0	11	2.21E−03
7	mtr-miR398a-5p	34	0	3	2.29E−03
8	PC-3p-54_81480	10,333	11,781	4,497	4.06E−03
9	lus-miR396b_R + 1_1ss19CT	691	1,088	665	5.88E−03
10	PC-5p-81557_56	26	3	18	6.20E−03
11	mtr-miR5284a_1ss9AT	31	3	16	8.79E−03
12	bra-MIR408-p5	5	0	6	8.82E−03
13	mtr-MIR5249-p3_2ss20TC24CT	5	0	1	1.07E−02
14	lja-miR390a-3p	155	69	130	1.14E−02
15	nta-miR172c_R + 1	4	0	7	1.18E−02
16	PC-5p-19253_324	91	75	143	1.19E−02
17	gma-miR159a-3p_1ss21AT	672	943	629	1.20E−02
18	cst-MIR11334-p5_2ss8TG17TC	0	6	3	1.40E−02
19	mtr-MIR156e-p3	89	316	62	1.41E−02
20	gma-miR171j-5p	63	19	64	1.54E−02
21	mtr-miR396a-5p	95,428	72,768	71,337	1.84E−02
22	mtr-miR5559-5p	1,655	810	156	1.98E−02
23	gma-MIR10428-p5_2ss14AG21AG	4	0	1	2.21E−02
24	mtr-MIR2111n-p3	50	120	56	2.27E−02
25	mtr-MIR2606a-p5_1	156	96	151	2.28E−02
26	mtr-MIR2606a-p5_2	156	96	151	2.28E−02
27	han-miR3630-3p_L + 3R-1_1ss24TA	4	68	29	2.43E−02
28	vvi-miR3630-3p_L + 3R-1_1ss5TG	4	68	29	2.43E−02
29	mtr-miR398b	265	106	44	2.45E−02
30	mtr-miR1507-3p	1,233	941	862	2.67E−02
31	PC-3p-102950_40	5	9	0	2.75E−02
32	mtr-miR5213-5p	2,132	1,529	1,239	2.82E−02
33	mtr-miR156b-5p	1,680	2,061	1,131	2.93E−02
34	gma-MIR1527-p3_2	13	4	1	3.36E−02
35	gma-MIR1527-p5_1	13	4	1	3.36E−02
36	gma-MIR1527-p3_1	13	4	1	3.36E−02
37	mtr-miR2675_L + 3_2ss4CT24TC	3	1	6	3.58E−02
38	mtr-miR2111l	465	1,098	799	3.62E−02
39	lja-MIR11078a-p3_2ss6GC18AT	4	14	9	3.65E−02
40	gma-MIR5368-p5_1ss1TC	3,421	10,041	10,476	3.68E−02
41	mtr-miR408-3p	5,082	3,537	1,264	3.80E−02
42	gma-MIR1527-p5_1ss13CA	23	12	25	4.10E−02
43	peu-MIR2916-p3_2ss5AG20TG	607	1,702	1,767	4.14E−02
44	mtr-MIR1510b-p5_1ss22CT	921	179	411	4.25E−02
45	mtr-miR5232	1,602	1,099	679	4.27E−02
46	PC-3p-61527_82	7	4	14	4.31E−02
47	mtr-MIR2603-p5_1ss24AG	142	78	158	4.55E−02
48	mtr-MIR2603-p3_1ss24AG	142	78	158	4.55E−02
49	ptc-miR162a_R + 1	38	21	18	4.55E−02
50	csi-miR167c-3p_L + 2R-2	156	76	100	4.73E−02

### Degradome Sequencing Analysis

Generally, miRNAs inhibit protein synthesis either by translational repression and/or mRNA target degradation (Eulalio et al., 2008; Filipowicz et al., 2008; Chekulaeva and Filipowicz, 2009). To analyze the relationships between the expression of miRNAs and mRNAs, miRNA targets were identified through degradome sequencing. After removing reads without adaptor sequences, 4,532,755, 5,691,194, and 6,414,386 unique mappable reads were obtained after filtering 4,574,314, 5,745,686, and 6,473,097 unique raw reads from the three degradome libraries (CK, DS, and RW), respectively. Then, 3,352,045, 4,295,738, and 4,516,291 unique *M. ruthenica* transcriptome sequences were mapped to the assembled transcriptome sequences, respectively. Transcriptome sequencing for 52,457 transcripts was conducted to detect *M. ruthenica* miRNA cleavage sites. Of these transcripts, 33,971, 34,304, and 37,758 (64.76, 65.39, and 71.98% of the input transcripts) were mapped to 18,279,670, 16,620,613, and 14,184,792 degradome reads, resulting in 86.29, 82.63, and 77.01% mapping ratios, respectively (Supplementary Table 7).

For data analysis, CleaveLand4 was used to identify sliced miRNA targets detected in our study. The abundance of raw tags was plotted for each target transcript, and the cleaved target transcripts were classified into five categories (0, 1, 2, 3, and 4) according to the abundance of tags at the site position of target transcript. The numbers of cleaved target transcripts in categories 0–4 were 115, 33, 1,720, 958, and 2,260 in CK library, 97, 44, 2,178, 1,284, and 2,716 in DS library, and 108, 36, 2,234, 1,267, and 2,866 in RW library, respectively (Supplementary Table 8).

### Identification of miRNA Targets *via* Degradome Sequencing in *M. ruthenica*

We obtained 11,390 miRNA-target pairs from the degradome sequencing of three libraries totally (Supplementary Table 8). AllenScore reflects the penalty of mismatched bases between the miRNA and its target, and is an important measure to evaluate the matching rate between the miRNA and its target (Addo-Quaye et al., 2009; Liu et al., 2016). In our study, analysis to degradome sequencing (AllenScore < 4) showed that 104 miRNAs (11 novel and 93 conserved miRNAs) and 263 target transcripts were identified, forming 296 miRNA-target pairs in three libraries (Supplementary Table 9). It was significantly seen that the maximum targets were obtained for ppe-MIR169i-p5_2ss17GT19TG, which had 90 target transcripts in three libraries. We inferred that ppe-MIR169i-p5_2ss17GT19TG may play important roles in *M. ruthenica*.

**FIGURE 4:**
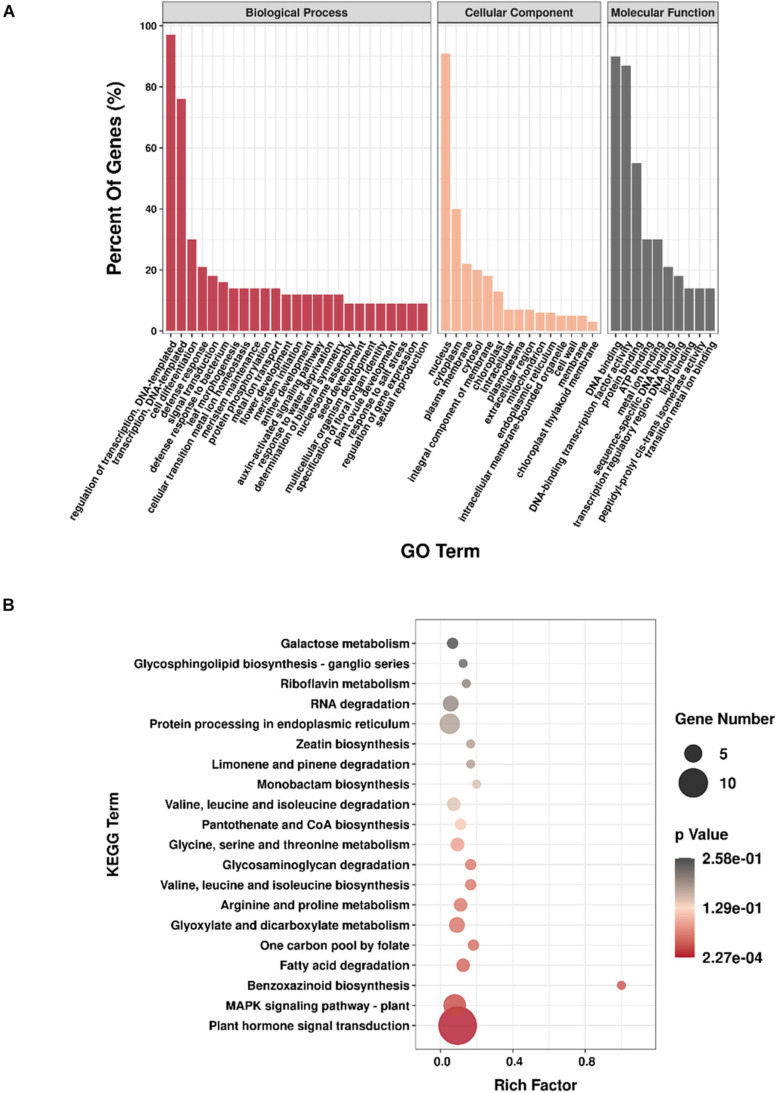
Enrichment analysis of target genes. **(A)** GO annotation of the genes targeted by miRNAs; **(B)** KEGG enrichment of genes targeted by miRNAs.

In total, 200 genes, targeted by the identified miRNAs, were annotated through GO analysis in the three libraries (Supplementary Table 10). Among the biological processes, “regulation of transcription, DNA-templated” and “transcription, DNA-templated” were the most abundant. For cellular components, the most frequent categories were “nucleus” and “cytoplasm,” and “nucleus” was also the most enriched group of all GO categories. Among the molecular function categories, “DNA binding,” “DNA-binding transcription factor activity,” and “protein binding” were the most abundant (Figure 4A).

**FIGURE 5:**
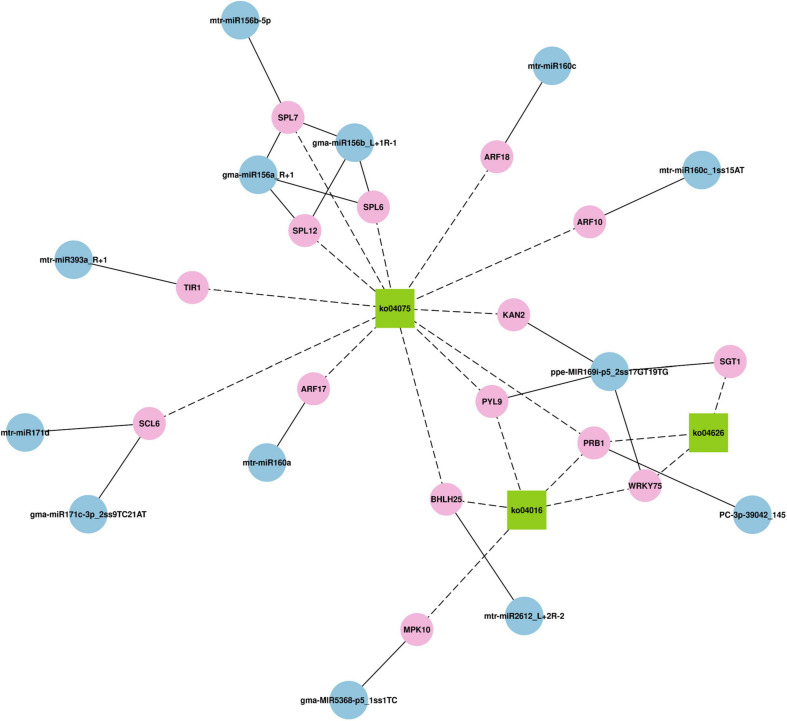
The network plot for the miRNAs and their targets associated with ko04016 (MAPK signaling pathway – plant) pathways, ko04075 (plant hormone signal transduction), and ko04626 (plant–pathogen interaction).

**FIGURE 6:**
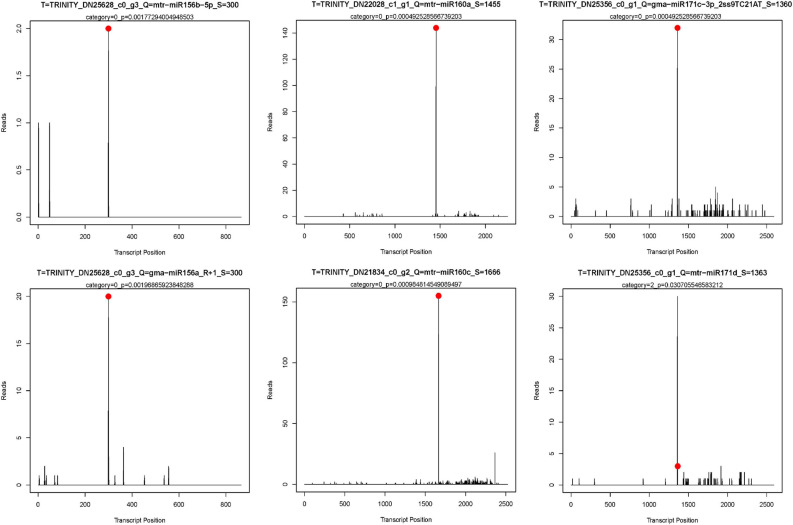
The target plots (T-plots) for target genes of miRNAs by degradome sequencing. *X*-axis, the site position of target transcript. *Y*-axis, the normal abundance of raw tags. Red circle, the cleavage site.

Then, through KEGG analysis, 78 targets were annotated to 67 different pathways (Supplementary Table 10). ko04075 (plant hormone signal transduction) and ko04016 (MAPK signaling pathway – plant) pathways, which were associated with 14 and 7 unigenes, respectively, were the most abundant pathways (Figure 4B), indicating their significant roles response to drought stress in *M. ruthenica*. Figure 5 is the network plot for the miRNAs and their targets associated with ko04016, ko04075, and ko04626 (plant–pathogen interaction) pathways. The squamosa promoter binding protein-likes (SPLs) targeted by miR156, and auxin response factors (ARFs) targeted by miR160 were mainly involved in plant hormone signal transduction. The PRB1 (putative reverse transcriptase, RNA-dependent DNA polymerase) targeted by the novel miRNA PC-3p-39042_145 was related to ko04016, ko04075, and ko04626 pathways. The PYL9 (putative polyketide cyclase/dehydrase, START-like domain-containing protein), which was the target gene of ppe-MIR169i-p5_2ss17GT19TG, was related to ko04016 and ko04075 pathways. Probable WRKY transcription factor 75 (WRKY75), which was the target gene of ppe-MIR169i-p5_2ss17GT19TG, was involved in ko04016 and ko04626 pathways. Several target plots (T-plots) for these miRNAs and their target genes validated by degradome sequencing were showed in Figure 6.

**FIGURE 7:**
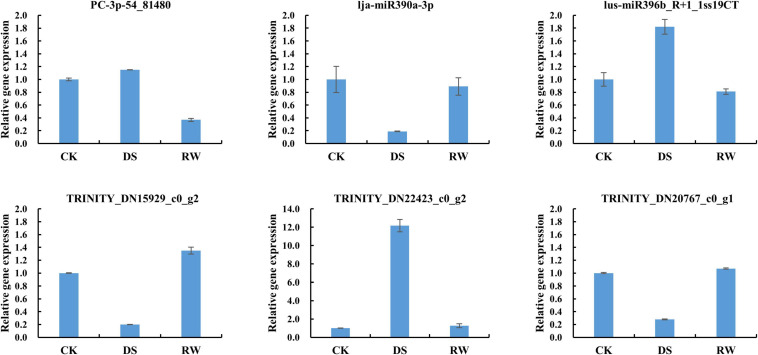
Relative expression results of miRNAs and their target *via* qRT-PCR in *M. ruthenica*. *GAPDH* of alfalfa and mtr-U6 were used as an internal reference for mRNA and miRNAs, respectively. All qRT-PCR reactions were repeated three times for each sample. Error bars indicate the standard deviation of three biological replicates.

Three miRNA-target pairs with negative regulatory relationships were chosen for validation *via* qRT-PCR analysis. The qRT-PCR results were consistent with those of Illumina sequencing and confirmed that the three miRNA-target pairs all displayed reversed expression pattern in our study (Figure 7). This also suggests that our small RNAs and degradome sequencing data are reliable.

### Correlation Analysis of miRNA Expression Profiles and Their Targets

Based on the degradome sequencing, we integrated the expression profiles of the miRNA and target genes from the different comparison groups, and obtained a table that summarizes the miRNA-target gene association analysis. After extracting information from the general table, we formulated a differential miRNA-differential target gene association analysis table, from which we obtained miRNA-target gene pairs with negative regulatory relationships. Corresponding to the DEMs, there were 38 differentially expressed targets from 16 miRNAs in DS vs. CK, 31 from 11 miRNAs in DS vs. RW, and 6 from three miRNAs in RW vs. CK (Supplementary Table 11). Then, 21, 18, and 3 miRNA-target gene pairs showed a reverse expression pattern in the DS vs. CK, DS vs. RW, and RW vs. CK comparison groups, respectively (Table 7). We found that gma-miR171j-5p was significantly downregulated in DS vs. CK and DS vs. RW, while its target, TRINITY_DN16578_c0_g1, was significantly upregulated. This may imply that the miRNA-target gene pair were specifically triggered by water deficit.

**TABLE 7:** The miRNA-target gene pairs showed reverse expression patterns in the three comparison groups.

Comparison	miRNA name		Target ID		Annotation
DS vs. CK	gma-miR171j-5p	↓	TRINITY_DN16578_c0_g1	↑	Uncharacterized protein LOC25496852 (*Medicago truncatula*)
	gma-miR171j-5p	↓	TRINITY_DN17512_c0_g1	↑	Kunitz-type elastase inhibitor BrEI (*Medicago truncatula*)
	lja-miR390a-3p	↓	TRINITY_DN22423_c0_g2	↑	Probable protein phosphatase 2C 2 (*Medicago truncatula*)
	mtr-MIR2118-p5	↓	TRINITY_DN16120_c0_g1	↑	Scarecrow-like protein 21 (*Medicago truncatula*)
	mtr-MIR2118-p5	↓	TRINITY_DN24512_c1_g1	↑	Putative fructose-bisphosphate aldolase (*Medicago truncatula*)
	mtr-MIR5230-p3_1ss3AG	↓	TRINITY_DN14908_c0_g1	↑	UV-B-induced protein At3g17800, chloroplastic-like (*Medicago truncatula*)
	mtr-MIR5230-p3_1ss3AG	↓	TRINITY_DN17507_c0_g1	↑	NAD(P)H-quinone oxidoreductase subunit O, chloroplastic (*Medicago truncatula*)
	mtr-MIR5230-p3_1ss3AG	↓	TRINITY_DN20655_c0_g1	↑	Delta (8)-fatty-acid desaturase 2 (*Medicago truncatula*)
	mtr-MIR5230-p3_1ss3AG	↓	TRINITY_DN24927_c0_g2	↑	Heat shock protein 70 (*Medicago sativa*)
	mtr-MIR5282-p3	↓	TRINITY_DN13678_c0_g2	↑	Hypothetical protein TorRG33 × 02_038580 (*Trema orientale*)
	mtr-MIR5282-p3	↓	TRINITY_DN17421_c0_g5	↑	Cytochrome P450 71A26 (*Medicago truncatula*)
	mtr-miR2119	↓	TRINITY_DN21843_c0_g1	↑	Guanylate kinase 3, chloroplastic (*Medicago truncatula*)
	mtr-miR2119	↓	TRINITY_DN22862_c0_g2	↑	Serine decarboxylase (*Medicago truncatula*)
	mtr-miR396a-3p_L-1	↓	TRINITY_DN15362_c0_g3	↑	E3 ubiquitin-protein ligase RDUF2 (*Medicago truncatula*)
	mtr-miR396a-3p_L-1	↓	TRINITY_DN16444_c0_g2	↑	Crocetin glucosyltransferase, chloroplastic (*Medicago truncatula*)
	mtr-miR396a-5p	↓	TRINITY_DN15305_c0_g6	↑	WRKY transcription factor 56 (*Medicago sativa*)
	mtr-miR396a-5p	↓	TRINITY_DN20346_c0_g1	↑	Senescence/dehydration-associated-like protein (*Medicago truncatula*)
	mtr-miR396a-5p	↓	TRINITY_DN23197_c0_g1	↑	Scarecrow-like protein 33 (*Medicago truncatula*)
	mtr-miR396a-5p	↓	TRINITY_DN23804_c0_g2	↑	Heat shock protein 83 (*Medicago truncatula*)
	mtr-miR396a-5p	↓	TRINITY_DN25757_c0_g1	↑	Nitrate reductase (NADH) (*Medicago truncatula*)
	mtr-miR398a-5p	↓	TRINITY_DN25174_c0_g2	↑	Probable solanesyl-diphosphate synthase 3, chloroplastic (*Medicago truncatula*)
DS vs. RW	PC-3p-102950_40	↑	TRINITY_DN19559_c0_g1	↓	Cytochrome P450 94B3 (*Medicago truncatula*)
	PC-3p-102950_40	↑	TRINITY_DN19590_c1_g1	↓	Putative heme peroxidase (*Medicago truncatula*)
	gma-MIR1527-p3_1ss4CT	↓	TRINITY_DN18346_c0_g4	↑	Uncharacterized protein LOC11435286 (*Medicago truncatula*)
	gma-MIR1527-p3_1ss4CT	↓	TRINITY_DN23107_c0_g2	↑	GDP-L-galactose phosphorylase 1 (*Medicago truncatula*)
	gma-miR159a-3p_1ss21AT	↑	TRINITY_DN20806_c0_g1	↓	UV-B-induced protein At3g17800, chloroplastic (*Medicago truncatula*)
	gma-miR171j-5p	↓	TRINITY_DN16578_c0_g1	↑	Uncharacterized protein LOC25496852 (*Medicago truncatula*)
	gma-miR171j-5p	↓	TRINITY_DN23921_c0_g1	↑	Fasciclin-like arabinogalactan protein 15 (*Medicago truncatula*)
	mtr-MIR2606a-p5_1	↓	TRINITY_DN15846_c0_g1	↑	Protein FAR1-RELATED SEQUENCE 5-like (*Medicago truncatula*)
	mtr-MIR5230-p3_2ss7TG19AG	↓	TRINITY_DN20517_c0_g2	↑	Proline dehydrogenase (*Medicago sativa*)
	mtr-miR156b-5p	↑	TRINITY_DN23829_c0_g2	↓	Cytochrome P450 78A3 (*Medicago truncatula*)
	mtr-miR168b	↑	TRINITY_DN17284_c0_g2	↓	Probable pectinesterase/pectinesterase inhibitor 34 (*Medicago truncatula*)
	mtr-miR172b	↓	TRINITY_DN14261_c0_g1	↑	Transcription factor bHLH35 isoform X1 (*Medicago truncatula*)
	mtr-miR172b	↓	TRINITY_DN14583_c0_g1	↑	Probable UDP-arabinopyranose mutase 1 (*Medicago truncatula*)
	mtr-miR172b	↓	TRINITY_DN16588_c0_g2	↑	Ethylene-responsive transcription factor RAP2-7 isoform X1 (*Medicago truncatula*)
	mtr-miR172b	↓	TRINITY_DN17480_c0_g1	↑	Uncharacterized protein LOC25483626 (*Medicago truncatula*)
	mtr-miR172b	↓	TRINITY_DN19871_c0_g1	↑	–
	mtr-miR172b	↓	TRINITY_DN20709_c0_g3	↑	Phototropic-responsive NPH3 family protein (*Medicago truncatula*)
	mtr-miR172b	↓	TRINITY_DN22378_c1_g1	↑	Probable methyltransferase PMT21 isoform X2 (*Medicago truncatula*)
RW vs. CK	mtr-MIR2592bj-p3_2ss12TC19AT	↑	TRINITY_DN21202_c0_g2	↓	Uncharacterized protein LOC25489828 (*Medicago truncatula*)
	mtr-MIR2603-p3_2ss9AC17AC	↑	TRINITY_DN22815_c0_g1	↓	lanC-like protein GCL1 (*Medicago truncatula*)
	mtr-miR396a-5p	↓	TRINITY_DN23804_c0_g2	↑	Heat shock protein 83 (*Medicago truncatula*)

*↑, upregulation; ↓, downregulation.*

## Discussion

Compared with other leguminous plants, little research has been conducted on the role of miRNAs in *M. ruthenica*. Here, three important high-throughput methods were applied to investigate the mechanism of drought resistance in *M. ruthenica*. The transcriptome data set was used as a reference sequence for small RNA and degradome sequencing analyses to identify the miRNAs and their target genes that might be associated with drought stress.

Through transcriptome sequencing, a total of 147,957 transcripts, assembled into 52,457 unigenes, were obtained from nine cDNA libraries. Through small RNAomics analysis, 532 conserved genes belonging to 65 miRNA families and 63 novel miRNAs were identified from miRNA libraries, 50 of which showed significantly different expression patterns among the three groups. Some miRNAs that responded to drought in previous studies were also detected in our results, such as miR156, miR159, miR162, miR171, miR396, miR398, miR408, miR1507, miR1510, miR2111, and miR3630 (Wang et al., 2011; Chen et al., 2015; Ferdous et al., 2015; Arshad et al., 2017; Li et al., 2017; Ji et al., 2018; López-Galiano et al., 2019), indicating the important roles of these conserved miRNAs under drought stress. We also found five novel miRNAs in the 50 DEMs, among which, PC-3p-54_81480 and PC-3p-102950_40 were upregulated under drought stress, and were downregulated after rewatering treatment; whereas the expression of PC-5p-19253_324, PC-3p-61527_82, and PC-5p-81557_56 were downregulated, and then upregulated in the aforementioned respective conditions.

Among the 50 DEMs, three members (mtr-miR398a-3p/a-5p/b) of the miR398 family and two members (bra-MIR408-p5 and mtr-miR408-3p) of the miR408 family were all downregulated under drought, which was consistent with the results in pea (Jovanović et al., 2014). In contrast, another study indicated that miR398a/b and miR408 were increased due to water deficit in *M. truncatula* (Trindade et al., 2010). These differences may be caused by the difference in species, extent and duration of drought stress (Wang et al., 2011), and sensitivity of some miRNAs to subtle differences in plant growth conditions (Ferdous et al., 2015). This suggests that even conserved miRNAs might function in a species-specific manner (Kamthan et al., 2015). In our study, some miRNAs from the same family (such as miR398, miR408, and miR1527) showed differently expressed trends after rewatering. Similar results were observed in rice, wherein members of miR319 showed different degrees of up- or downregulation responses to drought (Zhou et al., 2010). It is possible that the miRNA gene regulators change their expression after rewatering, leading to changes in miRNA expression patterns (Ferdous et al., 2015).

**FIGURE 8:**
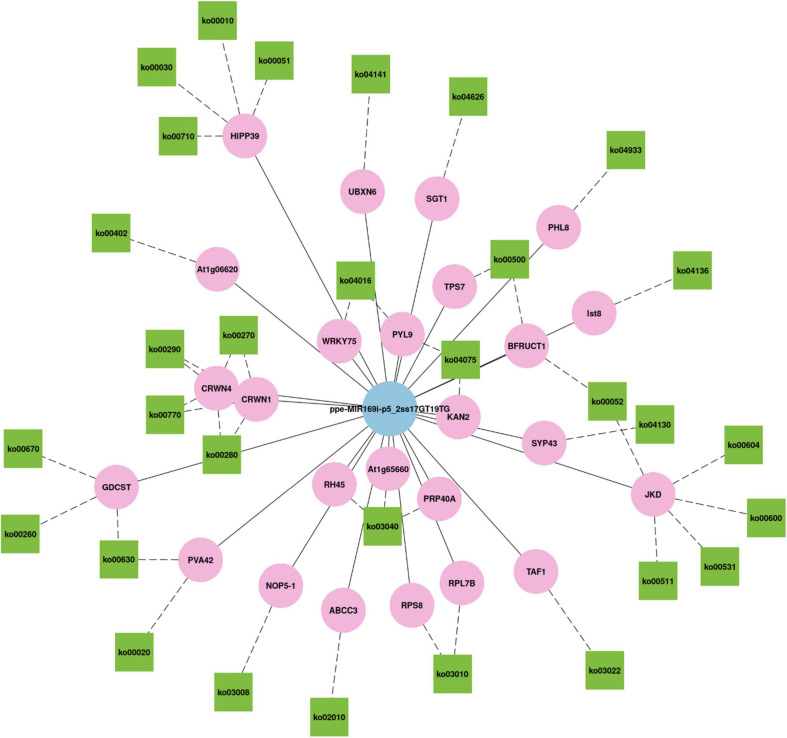
The network plot for the target genes of ppe-MIR169i-p5_2ss17GT19TG and their KEGG pathways.

Generally, a single miRNA can target several genes (Samad et al., 2017), and a single gene can be regulated by several miRNAs (Katiyar et al., 2015). We found that ppe-MIR169i-p5_2ss17GT19TG, which had the maximum number of targets among all miRNAs, targeted 90 transcripts in three libraries totally. miR169 is the largest miRNA family in *A. thaliana* and has 14 members, which can be divided into four groups according to their mature miRNA sequences: miRNA-169a, miRNA-169b/c, miRNA-169d/e/f/g, and miRNA-169h/i/j/k/l/m/n (Du et al., 2017). Asefpour Vakilian (2020) revealed the contribution of each miRNA to stress response in plants by implementing the feature selection algorithm on the constructed database. The algorithm showed that miRNA169 had the highest contribution to drought stress (Asefpour Vakilian, 2020). Li et al. (2008) also demonstrated that miR169a and miR169c were substantially downregulated due to drought stress, and that miR169a mainly regulates *NFYA5* expression at the mRNA level (Li et al., 2008), while miR169i mainly affects *NFYA5* expression at the translational level (Du et al., 2017). In our study, a total of 78 ppe-MIR169i-p5_2ss17GT19TG targets were annotated by GO terms in three libraries (Supplementary Table 12). The nucleus (GO:0005634) involving 24 genes was the most enriched group of all GO categories, followed by the cytoplasm (GO:0005737), protein binding (GO:0005515), and plasma membrane (GO:0005886). KEGG analysis of ppe-MIR169i-p5_2ss17GT19TG targets showed that 30 genes annotated to 33 different pathways (Supplementary Table 12). Figure 8 was the network plot for the target genes of ppe-MIR169i-p5_2ss17GT19TG and their KEGG pathways, for instance, CRWN (protein CROWDED NUCLEI) involved in ko00270 (Cysteine and methionine metabolism), ko00280 (Valine, leucine, and isoleucine degradation), ko00290 (Valine, leucine, and isoleucine biosynthesis), and ko00770 (Pantothenate and CoA biosynthesis) pathways simultaneously. JKD (zinc finger protein JACKDAW) related to ko00052 (Galactose metabolism), ko00600 (Sphingolipid metabolism), ko00531 (Glycosaminoglycan degradation), ko00604 (Glycosphingolipid biosynthesis – ganglio series), and ko00511 (Other glycan degradation) pathways, which revealed the extensive regulatory roles of ppe-MIR169i-p5_2ss17GT19TG in *M. ruthenica*.

Degradome sequencing is based on high-throughput sequencing technology and bioinformatics analysis and avoids false positive results effectively, which makes it more suitable for plant miRNA target gene identification (Sun et al., 2015). Through the correlation analysis of the transcriptome-small RNA-degradome, we obtained differential miRNA-differential target gene association results and miRNA-target pairs with negative regulatory relationships. Among them, two miRNA-target pairs appeared in two comparison groups. gma-miR171j-5p was significantly downregulated in DS vs. CK and DS vs. RW, while its target TRINITY_DN16578_c0_g1 was significantly upregulated. Similarly, mtr-miR396a-5p was significantly downregulated in DS vs. CK and RW vs. CK, while its target TRINITY_DN23804_c0_g2 was significantly up-regulated. *SCL6-II* and *SCL6* are the target genes of miR171 in *A. thaliana* (Lee et al., 2008), which play roles in plant root and leaf development, photochrome signaling, lateral organ polarity, meristem formation, vascular development, and stress response (Wang et al., 2010; Zhu et al., 2015; Asefpour Vakilian, 2020). gma-miR171o and gma-miR171q regulate *GmSCL-6* and *GmNSP2*, respectively, which in turn influence the spatial and temporal aspects of soybean nodulation (Hossain et al., 2019). miRNA-396 is an important contributor to the plant stress response which targets four classes of stress resistance proteins: pathogen-related, nucleotide binding site resistance protein-like, dirigent-like, and ribonuclease-like proteins (Zhang et al., 2007). miRNA-396 targets cell cycle regulators, as well as those that control plant growth and differentiation. miRNA-396 is increased under stress and represses cell multiplication (Patel et al., 2019).

## Conclusion

In conclusion, we elucidated the small RNAs and their target genes in *M. ruthenica* when subjected under drought stress and rehydration treatment though transcriptome, small RNA, and degradome sequencing. Although the complex miRNA-mediated regulatory networks remain to be elucidated, these findings provide valuable information for further functional characterization of genes and miRNAs in response to abiotic stress, in general, and drought stress in *M. ruthenica*. More importantly, this study will serve as a foundation for future research on the functional roles of miRNAs and their target genes in legume forage.

## Materials and Methods

### Plant Material and Drought Treatments

*Medicago ruthenica* seeds were obtained from the Institute of Grassland Research, Chinese Academy of Agricultural Sciences at the Inner Mongolia Autonomous Region, China. This cultivar grows well in Inner Mongolia, with higher drought and cold resistance. The sanded *M. ruthenica* seeds were sterilized in concentrated sulfuric acid for 10 min, and then washed thoroughly with sterile water (Araújo et al., 2004). After incubating for 3 days at 4°C in dark conditions, the seeds were placed on soaked filter paper in Petri dishes, and incubated in an artificial climate chamber (temperature, 25 ± 2°C; photoperiod, 16 h light/8 h dark cycle; relative humidity, 50%). Two days later, the germinated seeds were transferred to pots (13 cm in diameter and 10 cm deep) with vermiculite and quartz sand. There were eight plants in each pot. Four-week-old seedlings were randomly divided into three groups: the control group (CK), the drought stress treatment group (DS), and the rewatering group (RW). The drought treatment period lasted for 15 days. During the experiment, CK plants were watered every 3 days, maintaining a relative soil moisture content of more than 80%, while DS and RW plants were subjected to water deprivation for 15 days, resulting in a relative soil moisture content of 20% on the 15th day. The RW plants were rehydrated to full soil saturation on the 15th day. To avoid any interference, no nutrients were added. Other growth conditions were maintained. Each treatment group had three biological replicates. Leaf samples of CK and DS were collected on the 15th day, and leaves of RW were collected 2 days after rewatering. Samples were immediately frozen in liquid nitrogen, and stored at −80°C for later use.

### Transcriptome Libraries Construction, Sequencing, and Analysis

Total RNA was isolated from *M. ruthenica* leaves using TRIzol reagent (Invitrogen, CA, United States) according to the manufacturer’s protocol. Total RNA quantity and purity were analyzed using a Bioanalyzer 2100 (Agilent, CA, United States) and RNA 6000 Nano LabChip Kit (Agilent, CA, United States), respectively. Poly(A) RNA was obtained from total RNA (5 μg) using poly T oligo-attached magnetic beads after two rounds of purification. Following purification, the mRNA was fragmented into small pieces using divalent cations at elevated temperatures. Then, the cleaved RNA fragments were reverse-transcribed to create the final cDNA library by following the protocol for the mRNASeq sample preparation kit (Illumina, San Diego, CA, United States). The average insert size for the paired-end libraries was 300 bp (±50 bp). Paired-end sequencing was performed using an Illumina Hiseq4000 instrument (LC Sciences, United States) according to the manufacturer’s instructions.

The adaptor contamination, low quality bases, and undetermined bases from raw data were removed using Cutadapt (Martin, 2011) and perl scripts in house. Then sequence quality was verified by FastQC,^7^ including the Q20, Q30 and GC-content of the clean data. All downstream analyses were based on clean data of high quality. *De novo* assembly of the transcriptome was performed with Trinity 2.4.0 (Grabherr et al., 2011). Trinity groups transcripts into clusters based on shared sequence content. Such a transcript cluster is very loosely referred to as a “gene.” The longest transcript in the cluster was chosen as the “gene” sequence (aka Unigene).

### Differentially Expressed Genes Analysis

Salmon (Patro et al., 2017) was used to perform expression levels for genes by calculating TPM (Mortazavi et al., 2008). Statistically significant (*p*-value < 0.05) DEGs with a log_2_ (fold change) > 1 or log_2_ (fold change) < −1 we selected using the R package edgeR (Robinson et al., 2010). Next, GO and KEGG enrichment analyses were performed on the differentially expressed genes using in-house Perl scripts. The GO project is a bioinformatics resource on gene products and descriptions of functions (Gene Ontology Consortium et al., 2013). It creates annotations to describe the biological roles of individual gene products (e.g., genes, proteins, ncRNAs, complexes) by classifying them (Gene Ontology Consortium, 2015). The relationships between a gene product/or gene-product group to biological process, molecular function, and cellular component are one-to-many. GO ontologies can also use for annotation of gene-expression data (Gene Ontology Consortium et al., 2000). KEGG is a knowledge base for systematic analysis of gene functions in terms of the networks of genes and molecules (Ogata et al., 1999). KEGG is widely used for analyzing genomics, transcriptomics, proteomics, glycomics, metabolomics, and other high-throughput data (Kanehisa et al., 2017). The online Blast algorithm^8^ can be used to analyze genes. If the significant similarities of Blast lead to the assignment of designated enzymes, these genes can be tagged in the corresponding KEGG pathways (Altermann and Klaenhammer, 2005).

### Small RNAs Sequencing and Bioinformatics Analysis

Nine small RNA libraries were constructed from approximately 5 μg of total RNA using TruSeq Small RNA Sample Prep Kits (Illumina, San Diego, CA, United States). Then the libraries were sequenced by Illumina Hiseq 2500 (LC Sciences, United States) following the vendor’s recommended protocol.

The adapter dimers, junk, low complexity, repeats, and common RNA families (rRNA, tRNA, snRNA, and snoRNA) were removed from raw reads using an in-house program, ACGT101-miR (LC Sciences, Houston, TX, United States). Subsequently, unique sequences (18–25 nt) were mapped to specific species precursors in miRBase 22.0 through a BLAST search; conserved miRNAs and novel 3p- and 5p-derived miRNAs were then identified. Length variation at both 3′ and 5′ ends and one mismatch inside of the sequence were allowed in the alignment. The unique sequences mapping to specific species mature miRNAs in hairpin arms were identified as known miRNAs. The unique sequences mapping to the other arm of known specific species precursor hairpin opposite to the annotated mature miRNA-containing arm were considered to be novel 3p- and 5p-derived miRNA candidates. The remaining sequences were mapped to other selected species precursors (with the exclusion of specific species) in miRBase 22.0 by BLAST search, and the mapped pre-miRNAs were further BLASTed against the specific species genomes to determine their genomic locations. The above two we defined as known miRNAs. The unmapped sequences were BLASTed against the specific genomes, and the hairpin RNA structures containing sequences were predicated from the flank 120 nt sequences using RNAfold software.^9^

### Detection of Differential Expressed miRNAs

The differential expression of miRNAs, based on normalized deep-sequencing counts, was analyzed by *T*-test. The criteria to classify DEMs between two-way pairing among the three experimental conditions (CK vs. DS; DS vs. RW; RW vs. CK) were as follows: the significance thresholds were set to be *p* < 0.05 in each test.

### Construction and Analysis of Degradome Libraries

The degradome libraries construction was further optimized and simplified based on Ma et al. (2010). Total RNA from the three groups (CK, DS, and RW) was isolated and purified using TRIzol reagent (Invitrogen, CA, United States) according to the manufacturer’s protocol. The RNA amount and purity of each sample were quantified using a NanoDropND-1000 (NanoDrop, Wilmington, DE, United States). RNA integrity was assessed using Agilent 2100 with RIN number > 7.0. Poly(A) RNA was purified from the plant total RNA (20 μg) twice using poly T oligo-attached magnetic beads. Because the three RNA cleavage products contain a 5′-monophosphate, the 5′ adapters were ligated to their respective 5′ ends using RNA ligase. Then, the first strand of cDNA for each mRNA was reverse transcribed using a 3-adapter random primer. Size selection was then performed using AMPureXP beads. Afterwards, the cDNA was amplified *via* PCR under the following conditions: initial denaturation at 95°C for 3 min; 15 cycles of denaturation at 98°C for 15 s, annealing at 60°C for 15 s, and extension at 72°C for 30 s; then a final extension at 72°C for 5 min. The average insert size for the final cDNA library was 200–400 bp. Lastly, we performed 50 bp single-end sequencing using an Illumina Hiseq2500 (LC Bio, China) according to the manufacturer’s recommended protocol.

Raw reads were subjected to ACGT101-DEG (LC Sciences, Houston, TX, United States) for data processing. This program mainly depends on CleaveLand4, a public software package for analyzing “degradome” data. Degradome data are a variant type of RNA-seq data, where the reads derive from the 5′-ends of uncapped RNAs (German et al., 2008; Addo-Quaye et al., 2009). These data can be used to identify miRNA and siRNA targets that are actively “sliced.” CleaveLand4 handles several phases of degradome data analysis in a single command, including: Alignment of degradome data to the reference transcriptome, and parsing the output into a “degradome density file.” The degradome density file reflects the counts of 5′ positions across the transcriptome. Alignment of query miRNAs or siRNAs to the transcriptome to generate a list of potential target sites. This uses the program “GSTAr.pl” (Generic Small RNA Transcript Aligner), which ships with the CleaveLand4 program. GSTAr.pl uses RNA–RNA thermodynamic predictions instead of sequence similarity to identify potential target sites, making it much slower than generic aligners, but more sensitive in terms of finding all possible sites. Cross-referencing the degradome data with the alignments to identify slicing sites with evidence of slicing. This includes assessment of *p*-values.

All the identified target genes were annotated *via* GO (see text footnote 1) and classified using KEGG pathways (see text footnote 2).

### Verification of mRNAs and miRNAs *via* Quantitative Real-Time PCR

The RNA-seq results for both mRNA and miRNA expression were verified *via* qRT-PCR. Total RNA was extracted from nine leaf samples using TRIzol reagent (Invitrogen, CA, United States). For the miRNA expression analysis, the U6 snRNA of *M. truncatula* was used as the reference gene. The alfalfa GAPDH gene was used as an internal reference for the mRNA expression analysis. All primers used for qRT-PCR are listed in Supplementary Table 13. qPCR was conducted using the ChamQ SYBR Color qPCR Master Mix (2X) on a LineGene9600plus Real Time PCR instrument. Three biological and three technical replicates were performed for each sample. Relative expression levels were calculated using the 2^–ΔΔCo^ method (Livak and Schmittgen, 2001).

## Data Availability Statement

The datasets presented in this study can be found in online repositories. The names of the repository/repositories and accession number(s) can be found below: NCBI GEO and GSE169056.

## Author Contributions

FM and RS conceived and designed the study. RS, WJ, XZ, and YL conducted the experiments. All authors carried out the data analysis. RS wrote the manuscript. FM, WJ, LY, ZZ, XZ, and QW revised the manuscript. All authors contributed to the article and approved the submitted version.

## Conflict of Interest

The authors declare that the research was conducted in the absence of any commercial or financial relationships that could be construed as a potential conflict of interest.

## Publisher’s Note

All claims expressed in this article are solely those of the authors and do not necessarily represent those of their affiliated organizations, or those of the publisher, the editors and the reviewers. Any product that may be evaluated in this article, or claim that may be made by its manufacturer, is not guaranteed or endorsed by the publisher.

## Footnotes

1
http://www.geneontology.org

2
http://www.genome.jp/kegg/

3
http://pfam.xfam.org/

4
http://www.expasy.ch/sprot/

5
http://eggnogdb.embl.de/

6
http://www.ncbi.nlm.nih.gov/

7
http://www.bioinformatics.babraham.ac.uk/projects/fastqc/

8
http://www.ncbi.nlm.nih.gov/BLAST/

9
http://rna.tbi.univie.ac.at/cgi-bin/RNAfold.cgi
